# Is There a Need for Cultural Adaptation of the Last Aid Course?—A Mixed-Methods Study across the Danish-German Border

**DOI:** 10.3390/healthcare10040658

**Published:** 2022-03-31

**Authors:** Georg Bollig, Mariam Safi, Marina Schmidt, Hermann Ewald

**Affiliations:** 1 Department of Anesthesiology, Intensive Care, Palliative Medicine and Pain Therapy, HELIOS Klinikum, 24837 Schleswig, Germany; 2 Internal Medicine Research Unit, Department of Regional Health Research University of Southern Denmark, University Hospital of Southern Denmark, 6400 Sønderborg, Denmark; mariam.safi2@rsyd.dk; 3 Last Aid International, 24837 Schleswig, Germany; 4 Letzte Hilfe Deutschland gGmbH, 24837 Schleswig, Germany; marina.schmidt@letztehilfe.info; 5 Katharinen Hospiz am Park, 24937 Flensburg, Germany; hermann.ewald@katharinen-hospiz.de

**Keywords:** Last Aid course, palliative care, public palliative care education, citizens, qualitative interview, mixed methods

## Abstract

Last Aid courses (LAC) have been established in 20 countries in Europe, Australia, and America to improve the public discourse about death and dying and to empower people to contribute to end-of-life care in the community. A mixed-methods approach was used to investigate the views of LAC participants about the course and cultural differences in relation to care and nursing at the end of life in the border region of Germany and Denmark. One-day workshops were held, including Last Aid courses in German and Danish, focus group interviews, and open discussions by the participants. The results show that almost all participants appreciate the LAC as an option to talk and learn about death and end-of-life care. The informants find individual differences more important than cultural differences in end-of-life care but describe differences connected to regulations and organization of services across the border. Suggestions for adaptation and improvement of the LAC include the topics of organization and support across the border, religions, and cultures, and supporting people in grief. The findings of the study will inform a revision of the Last Aid curriculum and future projects across the border and will help to include the views of minorities.

## 1. Introduction

Since 2015, Last Aid courses have been introduced in 20 different countries in Europe, Brazil, Australia, and Canada [1], and Last Aid International includes, at present, 20 countries worldwide. The Last Aid course (LAC) aims to educate people about palliative care and end-of-life care, seeks to enhance the public discourse about death and dying in general, and to improve people’s participation in end-of-life care provision at home [2,3,4,5,6]. A recent review summarized that Last Aid courses are both feasible and well accepted by people in different countries and with different cultural backgrounds and nationalities [1]. The courses can contribute to a public debate on death and dying and may contribute to empowering people to engage in end-of-life care [1]. Participation of the public in palliative care provision and end-of-life care and the formation of compassionate communities are important in order to secure appropriate support for the increasing numbers of people in need of palliative care at home in the future [6,7,8,9,10]. Previous studies have shown that most people wish to die in their own home and that over 60% of these require general palliative care [7,8,11]. General palliative care provision should be a cooperation of the healthcare services in the community (general practitioners and district nurses) and citizens. Public palliative care education and LAC may contribute to improving palliative care in the communities by providing knowledge and opportunities for discussing the subject of death and dying openly and can contribute to motivating and empowering citizens to engage in end-of-life care [1,3,4]. As LAC is currently implemented in different countries and regions of the world with people from different nationalities, ethnic and cultural backgrounds, the question arises if the LAC should be adapted in order to respect the needs of different cultures and ethnic groups, including minorities and indigenous people.

Therefore, the aim of the present study was to investigate the opinions and views of Last Aid course participants about the course and cultural differences in relation to care and compassion at the end of life in the border region of Germany and Denmark with minorities living on both sides of the border.

In order to reach this aim, people with a variety of cultural backgrounds living in the Danish-German border region were invited to participate in workshops and to be included as informants in the study. The results from the study are intended to inform a revision of the international Last Aid course curriculum and materials in order to make it suitable for people from different cultures and nationalities.

## 2. Materials and Methods

### 2.1. The Last Aid Course Concept

The international Last Aid course concept is based on a standardized curriculum grounded in a consensus of a multi-professional working group of international experts from the field of palliative care [1,2,3,4]. The idea of LAC was first described in 2008 by Bollig, and the courses have spread around the world after first experiences in Norway, Germany, and Denmark during the years 2014–2016 [1,2,3,4]. The LAC consists of four modules of 45 min each and is usually taught during one afternoon or evening in a classroom setting with 6 to 20 participants attending. The main themes of the four modules are: (1) dying as a normal part of life; (2) planning ahead; (3) relieving suffering; and (4) final goodbyes. The LAC consists of educational lectures, practical demonstrations, and practical training (e.g., measures to relieve pain, breathlessness, nausea, and information about locally available palliative care services and support) plus reflection on and discussion about death and dying in general [1,2,3,4]. The course includes, for example, themes such as end-of-life care, advance care planning and decision making, symptom management, burial, and cultural aspects of death and bereavement. Table 1 provides an overview of the Last Aid course modules and contents. All certified LAC instructors have experience in the field of palliative care and include nurses, physicians, social workers, priests, hospice volunteers, and others. The LAC must be held by a team of two certified instructors, one of whom must be either a nurse or a doctor working in palliative care [1,2,3,4].

### 2.2. Setting and Participants

**Figure 1:**
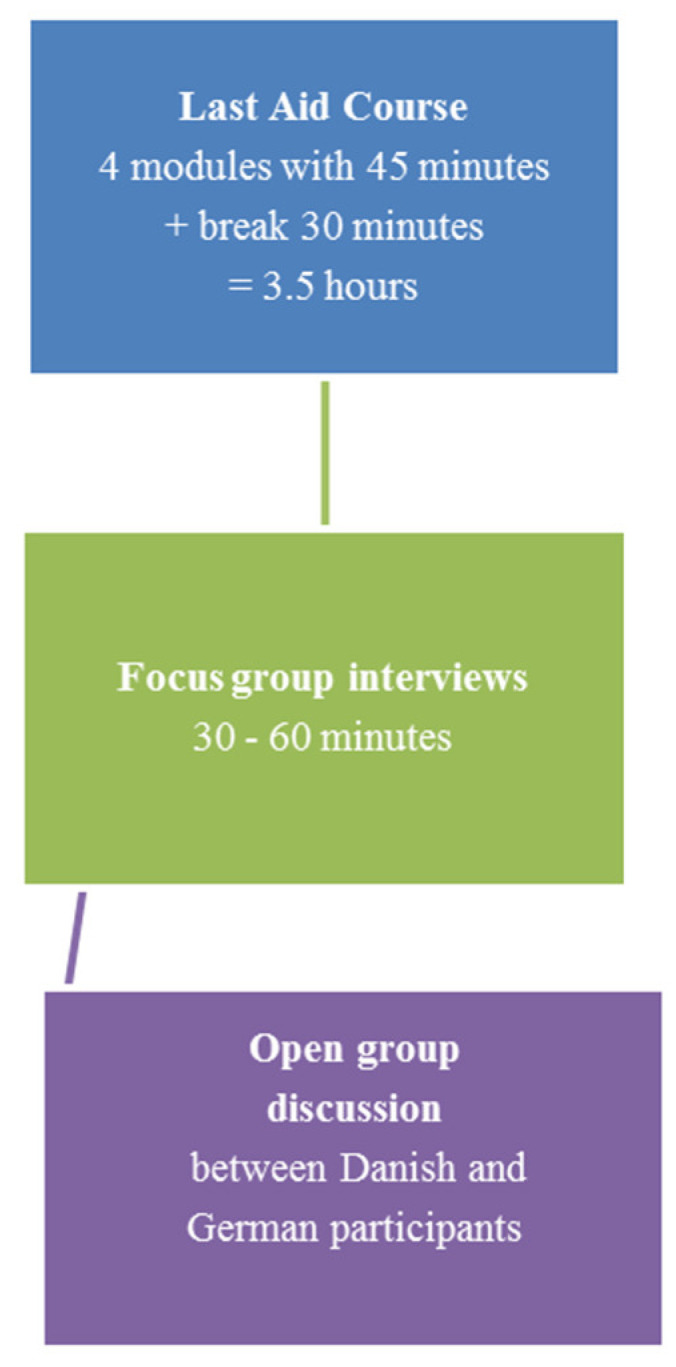
Overview of the one-day Last Aid workshops.

The first plans for the current project were made before the start of the worldwide COVID-19 pandemic. Before the COVID-19 pandemic started, three one-day workshops were planned to be held in the German-Danish border region. These workshops aimed to combine two groups, one in Danish and one in German. Both groups were planned to attend a normal LAC in the language of their choice (Danish or German). Afterward, the participants would be invited to join a focus group interview about their views on the Last Aid course and cultural aspects of end-of-life care. Thereafter, a joint meeting with open discussions among the two groups was planned. However, following the start of the pandemic, meetings with many people and classroom teaching were either very restricted or impossible to perform as planned. Therefore, Last Aid courses in the present study were held in a classroom setting or online due to varying restrictions connected to the COVID-19 pandemic. Material for practical training was sent to the participants of the online LAC prior to the online course. Participants were recruited via a network of cooperating and supporting organizations in Denmark and Germany. Invitations to participate were published in the media using different information channels such as newspapers, radio, and social media channels, including Instagram, Facebook, and podcasts. All interested people were invited to participate and comprised of laypersons without experience in palliative care, experts from the field of palliative care, and representatives from different organizations. The workshops usually lasted one day, including a normal LAC in the morning and a focus group interview in the afternoon, followed by the opportunity to participate in a joint open discussion between the German and Danish participants. Figure 1 provides an overview of the one-day Last Aid workshops.

Purposive sampling was used for data collection. All participants of the workshops and Last Aid courses were asked to provide their feedback using a standardized questionnaire. All people who had participated in the one-day workshops and Last Aid courses were invited as informants for our study without any other selection criteria. This approach was chosen to ensure a high number of informants with different nationalities, private, religious, social, and professional backgrounds to provide a broad picture of different views and opinions on end-of-life care and the Last Aid course.

### 2.3. Professional Background of the Authors

The professional background of the authors was as follows: GB was working both as a senior consultant in palliative medicine at the University Hospital of Southern Jutland in Sønderborg, Denmark, and as a clinical associate professor in palliative care at the University of Southern Denmark; since march 2022 he is a senior consultant in palliative medicine and pain therapy and the head of palliative medicine at the HELIOS Klinikum Schleswig, Germany; MSA is a PhD student at the University of Southern Denmark; MSC is a nurse specialized in palliative care and CEO of the non-governmental organization Letzte Hilfe Deutschland gGmbH in Schleswig, Germany; HE is is a medical doctor (MD) specialized in palliative medicine, and the medical director of the Katharinen Hospiz am Park gGmbH, Flensburg, Germany, an ecumenical center for hospice and palliative care, providing a palliative care unit as well as specialized palliative care for outpatients among other things.

### 2.4. Data Collection and Analysis

The study was based on a mixed-methods approach [12] with a combination of quantitative and qualitative data from a questionnaire and focus group interviews. The reason for using a mixed-methods approach was to provide a bigger picture and to be able to explore the informants’ views more in-depth via interviews. All participants were asked to provide their feedback using a questionnaire (in Danish or German). The questionnaire was an adapted version of a questionnaire that was used in a previous multicenter study with more than 5000 participants on the Last Aid course [13] and included questions on the participants’ views on the different modules and the course, a rating of the course modules on a four-point scale, four questions on the informants’ views on the course that could be answered with yes or no plus the possibility to provide comments in own words of the informants and information about the informant‘s age, sex and profession. The questionnaire is available in Danish and German from the first author on request. Descriptive statistics were used for the presentation of the quantitative data from the questionnaires. After having participated in the Last Aid course, all participants were invited to participate in focus group interviews. Two researchers with experience in qualitative research facilitated the focus group interviews. A semi-structured guide for the focus group interviews used the following introductory questions:

- What are your views on the Last Aid course in general?
- What is important for you in care at the end of life?
- What is important in your culture in end-of-life care?
- Do you have suggestions for cultural adaptation of the Last Aid course?
- Do you have suggestions for improvement of the Last Aid course?

The interviews were held in either a classroom setting or online as a video meeting, digitally recorded and transcribed verbatim by experienced researchers and trained secretaries. Qualitative content analysis and qualitative description were used to analyze the qualitative data from the interviews and the qualitative data from the questionnaire [14,15,16]. Qualitative description aims to provide a comprehensive description and a summary of experiences in everyday language and is the method of choice for a straightforward description of phenomena [14]. Qualitative description is a useful method in mixed-methods research [17] and was used here to provide rich and straightforward descriptions of the participants’ views in everyday terms. It is a qualitative method that is close to the data and less interpretive than other methods such as interpretive description or grounded theory.

Quantitative data included the variables described above. Descriptive statistics were used to describe the quantitative data from the questionnaires. Analysis of the qualitative data was based on the qualitative description and qualitative content analysis with an inductive approach and collection of data-derived themes [12,14,15,16,17]. GB, MSA, and HE analyzed the qualitative data by repeated reading of the transcripts and establishing preliminary codes from the material. Codes were thereafter discussed between all the authors and revised in three discussion rounds. As the last step of the analysis, GB, MSA, and HE checked the data again and questioned the findings using meta-positions. Finally, all authors agreed on the codes and themes found in the qualitative data material and the interpretation of the data. Data collection, analysis, and presentation of the qualitative data are based on the Consolidated criteria for reporting qualitative research guidelines (COREQ) [18]. Supplementary File S1 provides a detailed report in accordance with the COREQ guidelines and a complete checklist with all 32 items for reporting qualitative research.

### 2.5. Ethical Considerations and Ethics Approval

Informants in the study were asked to complete a questionnaire after participation in the Last Aid course. They were also invited to participate in a focus group interview. The present study is a part of an ongoing larger research project that aims to evaluate the effects of Last Aid courses and has been reported to the Regional Ethical Committee of Southern Denmark. The Regional Ethical Committee concluded that no formal application was required (The Regional Committees on Health Research Ethics for Southern Denmark; nr. 20182000-33). Informed consent was obtained from all participants who received information about the purpose of the study prior to participation. In order to protect privacy, no personal data other than age, sex, and professional education were collected. The informants could choose whether they wished to provide this information or not.

## 3. Results

Due to the COVID-19 pandemic, classroom teaching was restricted and partly impossible in some periods during the project period from January 1st to 31st of December 2021. Therefore, a number of courses and workshops were held online. In total, eight Last Aid courses were held on five different dates. 79 people participated in Last Aid courses and workshops between 20th May and 7th December 2021. A total of 38 participants attended a course/workshop in Danish and 41 in the German language. Participants could choose the language themselves and were not divided into groups according to nationality or citizenship. Some Danish participants preferred to participate in a German-speaking course and workshop.

### 3.1. Results from the Quantitative Data from the Questionnaire

A total of 53 of the 79 participants returned a questionnaire resulting in a return rate of 67%. Information on age was provided by 46 of 53 participants (87%). The informants’ age ranged from 34 to 81 years, with a median of 63 and a mean of 60 years of age. A total of 41% of the informants who provided information about their age were 65 years or older, whereas only three participants (7%) were under 40 years old. A total of 90% of the participants were female. A total of 30% of the informants who provided information about their profession had a medical background. Descriptive statistics were used for the presentation of the quantitative data from the questionnaires and the description of the used variables.

Table 2 shows the data from the participants and participants’ ranking of the Last Aid course content. Almost all participants rated the course contents as very good or good. A total of 96% of the participants would recommend the course to others.

### 3.2. Results from the Qualitative Data from the Focus Group Interviews and Questionnaire

**Figure 2:**
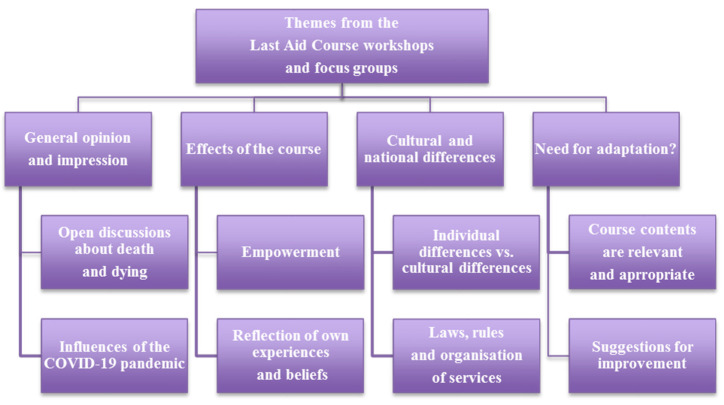
Themes from the Last Aid course workshops and focus groups.

A total of 49 of the 79 participants (62%) joined one of seven focus group interviews in Danish (three focus groups) or German (four focus groups). The analysis of the focus group interviews during the workshops led to four main themes and eight subthemes that will be described in more detail below (Figure 2). No predefined codes were used in the process of analyzing the qualitative data. The four main themes that are shown in the result section are connected to the research questions and the subthemes that emerged from the data material during the analysis of the interview transcripts. Data saturation [19] was reached for the qualitative data from the focus group interviews, including both data saturation for the data collection and inductive thematic saturation during the analysis of the qualitative data.

Quotations from the focus group interviews are provided to support each theme and are associated with anonymized codes for the informants. The codes for the informants provide the following information: date of the focus group interview, language (G for German or D for Danish), and participant number.

#### 3.2.1. General Opinion and Impression

Most participants appreciate the Last Aid course and its contents. Many informants find participation in the course meaningful and helpful. Many want to engage in future conversations about death and dying with other people and wish that these topics should receive more space in the public discourse.

My wish is that it (the topic of death and dying) had more space in the public… This has to be integrated into the society…

(11 September 2021, G1)

##### Open Discussions about Death and Dying

Participants’ experiences during the course are that it is both possible and meaningful to talk openly about death, dying, and care at the end of life. Many participants feel encouraged to talk about these issues with others after the course.

I think, what is important is, when you come back now and you have your circle of friends, that you simply talk about it (death and dying).

(18 August 2021, G6)

One important lesson learned for many participants is the recognition and acceptance that death is a normal part of life and not a catastrophic or avoidable event. A public debate about death and dying can also have an impact on the reflection of one’s own life and daily life, including the meaning of life and individual aims people would like to achieve during their lifetime.

When does the rest of your life start? It is also a question to discuss how you will spend the rest of your life?… and probably also to make decisions about my end of life…both things (life and death) belong together.

(18 August 2021, G1)

Some participants confirm that they gained new knowledge about death and dying in general by attending the Last Aid course.

I think it (the course) has given me more knowledge about everything that has to do with: what happens to those who are dying?; what happens with the relatives? And which natural mechanisms—both physical and mental- are happening in humans?

(11 September 2021, D2)

##### Influences of the COVID-19 Pandemic

The COVID-19 pandemic has led to major changes in public life, including many restrictions on meeting, working, and traveling. Many people died from COVID-19, and death may have been experienced much closer in their everyday life. The pandemic has also changed communication throughout society and has obviously had a great impact on the traditional Last Aid courses held in a classroom setting. Our informants stated that COVID-19 has made it much more complicated to accompany people. The pandemic has also led to a lack of the possibility to say farewell as needed.

…I have experienced funeral services within the corona year and have seen what is lacking if people are not able to say farewell

(5 June 2021, G6)

In order to respond to the need to talk about death and dying and to provide information about palliative care despite the pandemic, online Last Aid courses were established shortly after the start of lockdowns of public life. Although many participants stated that they would prefer classroom teaching, they appreciated online courses as an option in pandemic times.

The online format…I am astonished again and again at what is possible to communicate online…I would not have thought that this is possible…

(5 June 2021, G5)

Some participants think that online teaching is the best option for them as living in rural areas makes it complicated to attend classroom teaching.

For me, it (the online course) is the first choice. If I imagine travelling from the edge of Eastern Holstein to Flensburg, this is a long journey…I have recognized throughout the last months that I have attended more online training than I could participate in, in a classroom setting.

(5 June 2021, G3)

For others, online courses are experienced as strenuous and only acceptable as an emergency measure or a second-best option for teaching during a pandemic. A much-appreciated option to sustain practical training as a part of the online Last Aid course was sending material for practical training to the participants prior to the online course. This can support practical training despite teaching online.

The supplement by mail was good…the mouth care package…that has added something to this online course and has established other possibilities…that we could practice mouth care. That was good.

(5 June 2021, G6)

#### 3.2.2. Effects of the Course

Many participants stated that they feel encouraged to talk about death and dying, and many feel more prepared to encounter death and to participate in end-of-life care provision at home.

##### Empowerment

People feel strengthened to talk openly about death.

I am definitely strengthened to deal more openly with it (death and dying)… I often experience that older people interrupt me, because you do not talk about that, and change the topic…We just have been talking about children. To allow for their naturalness and assimilate it. Their curiosity, being allowed to ask questions. Yes, I think that is important.

(11 September 2021, G1)

Participants learn that there are many things they can do and that simple measures can have great importance in the care of the dying. Some people are motivated to start their own advance care planning and to talk to their family and friends about wishes for their end of life.

…I met my son outside, he was working in the garden, and I have told him that he shall be my representative in the future. That is what I have learned today, how important a representative is…who is able to take care of different issues…and I will contact a funeral bureau to find out how much that will cost, this interests me.

(20 May 2021, G2)

I have been inspired to try to write a letter to my two girls who are really close to me. They cannot stand to hear about my wishes (for the end of life and burial) because they do not want to face it.

(18 August 2021, D5)

Many participants recognized the need for public engagement and volunteering within palliative care and end-of-life care and that doing shopping for others can be a simple thing to do.

##### Reflection on Own Experiences and Beliefs

Many participants reflected on their own attitudes and beliefs connected to death and dying, and many experienced reflection on the meaning of life, spirituality, religious beliefs, and other philosophical questions as an effect of participating in the LAC.

The course was good because it encouraged me to reflect, in addition to providing information. It leads to reflection on how we handle things in our family and where we are coming from…if you have not experienced it (death) before you do not know how the process works.

(20 May 2021, D10)

One participant shared the story of her sister, who was able to talk about the death of her child for the first time after she had participated in the Last Aid course. This example indicates that the course may support the bereavement process for some people.

I have experienced how important this course was for my sister. She has participated in the (Last Aid) course two or three years ago in Hamburg. She had lost her four month old baby in 1979. After the course, she has talked about that for the very first time with me. Before she had repressed it. She ran towards the emergency doctor with her dead child…that was an awful experience. But after the course, she talked about it.

(18 August 2021, G10)

Many informants stated that they have changed their view on the importance of individuality and think that individual decisions should be more respected at the end of life. Respecting wishes is paramount in the dying phase, where respecting wishes can inform carers of what to do and how to care.

That one respects the wishes of the dying. These may be totally different. Some want contact and touch; others just want to be left in peace. That it is possible to find out what the person wants and what one should avoid doing although they (the Dying) are not able to talk anymore.

(18 August 2021, G4)

Interestingly, some participants attended the course after they had been involved in end-of-life care for others. Some participants stated that if they had participated in the course before they had to care for a dying person, they would have done things differently. Others describe it as a relief to hear that they did it right—as a sort of confirmation and relief that they did what they could to care for a dying person.

…to receive a confirmation that things that I have done were right. That one does not need to have a guilty conscience to have done something wrong. In my view, this is a conclusion from this event.

(18 August 2021, G7)

One participant described a very moving story about how they stayed with a dying person until the end and the very last breath.

We knew that my dad was going to die. And he was struggling to breathe…And then we told him: You are going to die. You do not need to breathe anymore. Then he became more relaxed and died an hour after. But he was really calm…We sang for him and everyone said farewell…Just say: You do not need to breathe anymore.

(18 August 2021, G7)

Other participants who had contributed to end-of-life care for someone stated that they wished that they had taken the course before they had to care for a dying person.

I must say that if I had taken the course a year ago… the situation when I had to care for my dying wife would have been different. Then I would have had other tools to use. I had to find out everything on my own…Our family doctor was not helpful at all…Yes, it was a good course…But it would have been good to know all these things a year ago.

(11 September 2021, D3)

#### 3.2.3. Cultural and National Differences

##### Individual Differences vs. Cultural Differences

Most informants state that they do not experience important cultural differences between Denmark and Germany. Many stated that there are important individual differences between different persons or families that need attention. The Danish participants had not experienced any cultural differences. They thought that most often, rituals related to death were very individual. Some participants described differences in organization and service within palliative care and levels of bureaucracy.

We are always confronted with questions that are not easy to answer. Fortunately, we do have a regional representative here in Padborg…I have his number and can ask questions about citizenship (rules and regulations).

(20 May 2021, G2)

What I have heard today is that there are some cultural differences but that I may ask the affected person or his relatives if I do not know…but I am able to be there as a human being and can look at what I might be able to contribute actively with.

(11 September 2021, G8)

Different rituals and customs are not only determined by nationality and religion but have many regional and individual aspects. Thus, it seems that they are more individually than culturally influenced.

I too think it is very much the same in Southern Jutland where we come from…If one dies at home, the neighbors set flowers on the road the hearse uses. If one dies in the hospital, the hearse will pass by one’s house to say farewell, if you may say so. But, I have never heard about that up in Northern Jutland, no, they do not know that (custom).

(11 September 2021, D4)

We sing and we accompany them (the deceased) on the way out. The more people participate the better it is. When the undertaker leaves the room (with the coffin) we stand in line.

(18 August 2021, G9)

One informant stated that it should not be too religious.

In my culture, for me, it is very important for me that it should not be religious.

(11 September 2021, G12)

Some participants mentioned the importance of mother tongue at the end of life and appreciated that many people are able to speak both Danish and German in the border region.

The German-Danish…the experience shows that many German-born speak in their mother tongue when they need care (at the end of life)

(20 May 2021, G2)

##### Laws, Rules, and Organization of Services

The organization of services for home care and palliative care shows vast and important differences between Denmark and Germany, for example, how often the home care service offers home visits per day. Often people have questions about different rules and services on both sides of the border.

My girlfriend is often here with me in Denmark but lives in Flensburg (Germany). That is a relationship across the border. We are always confronted with questions that are not easy to answer. Fortunately, we have a regional representative here in Padborg…I have his number and can ask questions about citizenship (rules and regulations).

(20 May 2021, G2)

#### 3.2.4. Need for Adaptation?

Most informants from both the Danish and German groups view the current Last Aid course as complete without the need for other themes, extensions of the contents, or timeframe. They prefer the course to stay as it is.

From my point of view, the Last Aid Course is very well balanced…when I imagine that it is made for normal citizens I think it covers everything needed without using technical language.

(5 June 2021, G3)

##### Course Contents Are Relevant and Appropriate

Most participants appreciate the course format with lectures, discussions, and practical training. Most of them view the course contents as informative, complete and do not have suggestions for additional content.

More could be too much. One is saturated with one day. Probably something comes up on the way home. But you can then read or ask about it, or talk to others.

(18 August 2021, G9)

Other suggestions from the participants were that LAC should be offered in the whole country and should become as normal as first aid courses.

We need Last Aid Courses. They should be as integrated as first aid courses.

(11 September 2021, G2)

##### Suggestions for Improvement

The informants have some suggestions for improvement and want more information and/or discussion on the following three topics: (1) Organization and support across the border; (2) Religions and cultures and (3) Supporting people in grief. One participant suggested that information about Last Aid could be spread through an application running on a smartphone. Some participants think that the course contents are appropriate but would also like to receive written information about the course contents.

I have no suggestions (to improve the course content) but I would like to get a handout, something that I could take home.

(11 September 2021, G1)

##### Information about organization and support across the border

In general, there is a need to inform the public about options for palliative care provision and support in the local community.

What I experience is that many relatives are not aware of where to get help, what is available and how to contact someone to support them…and that they do not need to do it alone, that one can do it as a team and not alone.

(11 September 2021, G3)

Some participants would like to receive more information about the option for support and palliative care services on both sides of the border. Both the Danish and German participants mentioned this. Information about the health services and available support is of great importance because many families have close relations across the border.

There are families that live on both sides of the border where there can be different laws and rules. Especially about things like “plejeorlov” (financiall supportted leave from work for caring relatives in Denmark) for example.

(18 September 2021, D8)

##### Information about Religions and Cultures

Some participants suggested including basic information about the most common religions.

I would appreciate information about the different religions, short and including basic information about other religions…It does not need to fill so much; just a short introduction.

(20 May 2021, D11)

Other participants highlight that there is a need for respect for individuality and different religions and that individuality might be more important than religion in general. This suggested that one should attend to the individual’s beliefs and wishes rather than assuming that a member of a particular religion follows a certain way of dying provided by his/her religion.

I think that dying is very individual and I believe that it is not possible to cover all religions, then you could take the whole day…but what I have to say is that one should be open-minded…to look, to hear because people can tell us something…although someone might be Muslim it is not clear if he practices his religion…we may ask the relatives…I think individuality not just present in religion but also in other areas there are individual differences (that need to be addressed)

(5 June 2021, G4)

##### Information about Supporting People in Grief

Fear and grief are themes that are mentioned by some participants as important. Some of them would like to provide these themes more space in the course. The informants would like to receive suggestions on how to behave in contact with grieving people.

The one who is grieving should not have to walk around in the streets begging for company or care. There should be presence of others. They should take initiative.

(18 August 2021, G6)

## 4. Discussion

The results from the qualitative data show that most participants appreciate the Last Aid course, its contents, and the opportunity to talk openly about death and dying. After participating in the course, many feel encouraged to talk about death and dying with others and feel strengthened to participate in end-of-life care provision in the community. For some informants, participation leads to reflection on their own experiences and attitudes connected to dying and palliative care. In some cases, the course led to an ability to talk about experiences with death that had occurred years ago. Most participants find the contents of the Last Aid course appropriate and fitting to their needs and expectations. Although most informants did not see important cultural differences, some had suggestions for improvement and adaptation of the Last Aid course to the national and regional particularities. These suggestions included more information on and/or discussion of the following three topics: (1) organization and support across the border; (2) religions and cultures; and (3) supporting people in grief. The informants from our study find individual differences more important than cultural or religious differences in end-of-life care. Some participants would like to receive more information about different religions and cultures. As the time for these topics is limited during the Last Aid course, other sources for information from brochures and educational material on the Internet, including written materials and videos, could be used as supplements and additional information [20,21]. In addition, several differences are connected to regulations and the organization of services across the border. Information about these differences should be taken into account for a revision and eventually regional adaptation of the Last Aid course contents.

The COVID-19 pandemic has influenced awareness of death and dying in the public and led to more video communication and online learning. Some informants rated online courses as their preferred option for participation in Last Aid courses. During the COVID-19 pandemic, online Last Aid courses were established in different countries such as Germany, Scotland, and Slovenia. The online courses have led to the participation of new groups, such as younger people who prefer Internet-based learning and meeting option and people caring for seriously ill relatives at home who can not leave their homes to participate in classroom education [1,22]. Most informants would prefer personal meetings, but some informants from the present study would prefer online learning. These findings may indicate that online courses should continue to be offered after the pandemic to provide Last Aid courses for the public for specific groups. Previous research has shown that there are two groups that especially appreciate online LAC: namely young people who are frequent Internet users and people who are in a situation caring for others at home [22]. This encourages organizations to continue to offer online LAC also after the pandemic ends.

The quantitative data from the current study show that the majority of the participants appreciate both the course and its contents and rate the course modules and content as very good or good (see Table 2). The high acceptance and appreciation of the Last Aid course are similar in all studies that have been undertaken. Both adults and children want to talk and learn about death and dying, and more than 92% would recommend the course to others [2,3,6,13,22]. In the current study, the informants had a median age of 63 years, 90% (43 of 48) were women, and 30% (13 of 43) had a medical profession. The high percentage of female participants is similar to a large multicenter study from Germany, Austria, and Switzerland with a percentage of 88% [13]. Currently, the caregiving role in families is still often associated with women, and the results indicate that many women who are caregivers want to be informed and prepared for end-of-life care. This sex disparity is similar to care in dementia and hospices [23,24]. Interestingly first experiences from Sweden have shown a higher percentage of male LAC participants [25]. This indicates that efforts should be made to interest more men in participating in Last Aid courses. This can, for example, be done by introducing Last Aid courses in professional education for police officers, a predominantly male profession, as already is ongoing in Scotland and Germany. Compared to previous research, a higher percentage of medical professionals participated in our study. That medical professionals participate in Last Aid courses has been described in different studies, with a percentage of medical professionals ranging from 27% in the first pilot study [3] to 9% in a multicenter study with 5469 participants from Germany, Switzerland, and Austria [13] whereas the number in present data is 30% of the participants. This shows that nurses and doctors also want to talk about death and dying. The reason for the participation of medical professionals could be both the lack of these topics in professional education or the wish to discuss death from a broader perspective. A recent pilot study has shown that many doctors and nurses are interested in an extended Last Aid course [26]. The results of the current study indicate that there is a need to respect and talk about cultural differences and individual wishes. The current results indicate that these can be addressed within the normal international Last Aid course curriculum without major changes needed. The most important method to address these challenges is to talk about the topic in the group of participants and to include the different perspectives within the group. Therefore, the Last Aid course instructors/facilitators should address these issues related to local situation, surroundings, and the participants‘ needs and experiences. In order to enable the instructors/facilitators to do that, additional training, reflection, and materials may be used [20,21].

Further research on the experiences, wishes, and needs of Last Aid course participants is needed to provide a richer picture of the need for cultural adaptation of Last Aid courses in other regions of the world. The Last Aid Research Group Europe (LARGE) will focus on this topic, which also will be addressed during the third International Last Aid Conference in October 2022 [27]. Another important topic for further research on Last Aid courses and end-of-life care is the sex discrepancy in attending LAC and the provision of layperson end-of-life care in the community. A pilot study on the experiences of caregivers of palliative patients who have participated in LAC is ongoing and will be completed soon.

## 5. Limitations

The most obvious limitation of the current study is its focus on just one region, the German-Danish border region, and the minorities living there. Furthermore, people from both sides of the border are mixed by working or shopping in the neighboring country or by family bonds. It might therefore be possible that former cultural specialties might have been mixed too and seemingly disappeared. Nevertheless, as the first study on end-of-life care and Last Aid courses across a border addressing different cultures and languages, this is a contribution to knowledge and a first step in studying this topic in different regions of the world. Therefore, similar studies should be undertaken in other regions and with the participation of people from different ethnic, national, and cultural backgrounds.

## 6. Conclusions

The results of the current study indicate that the Last Aid course curriculum and contents are suitable for a wide range of participants and can be used for people with diverse ethnic backgrounds. Individual values and wishes seem to be of greater importance than the cultural or national background. More studies on Last Aid courses connected to individual and cultural differences are needed from different bordering regions in the world.

## Figures and Tables

**Table 1:** The Last Aid course contents.

Module No.	Topic	Course Content
Module 1	Dying as a normal part of life	Welcome and introductions First aid and Last Aid What you can do to care The process of dying
Module 2	Planning ahead	Networks of support Making decisions Medical and ethical aspects Advance care planning Advance directive Medical power of attorney
Module 3	Relieving suffering	Typical problems and symptoms Caring/relieving suffering Nutrition at the end of life How to comfort
Module 4	Final goodbyes	Saying goodbye/final farewell rituals Funeral and various forms of burials Grieving is normal Grief and ways of grieving Questions, comments

**Table 2:** Data from the participants and participants’ ranking of the Last Aid course content from the questionnaire (*n* = 53).

Number of Returned Questionnaires		*n* = 53
**Age group**	18–64	27
	65+	19
	No information provided	7
**Sex**	Female	43
	Male	5
	No information provided	5
**Professio** **n**	Medical	13
	Non-medical	30
	No information provided	10
**Rating—Module 1:**	Very good	42
Dying as a normal	Good	10
part of life	Satisfactory	1
	Inadequate	0
**Rating—Module 2:**	Very good	39
Planning ahead	Good	13
	Satisfactory	1
	Inadequate	0
**Rating—Module 3:**	Very good	40
Relieving suffering	Good	12
	Satisfactory	1
	Inadequate	0
**Rating—Module 4:**	Very good	40
Final goodbyes	Good	11
	Satisfactory	2
	Inadequate	0
**Overall ranking of the course as a whole**	Very good	41
	Good	11
	Satisfactory	1
	Inadequate	0
**Be** **neficial for everyone**	Yes	51
	No	0
	No information provided	2
**Learned a lot of new things**	Yes	43
	No	8
	No information provided	2
**The contents were easy to understand**	Yes	51
	No	0
	No information provided	2
**I will recommend the course to others**	Yes	51
	No	0
	No information provided	2

## Data Availability

The data presented in this study are available in part on request from the corresponding author. The data are not publicly available due to privacy restrictions.

## Supplementary Materials

The following supporting information can be downloaded at: https://www.mdpi.com/article/10.3390/healthcare10040658/s1, Supplementary File S1: Report on accordance with the COREQ guidelines—checklist for reporting qualitative research.
