# Supplementary material for: Is There a Need for Cultural Adaptation of the Last Aid Course?—A Mixed-Methods Study across the Danish-German Border
Source: Healthcare (Basel). 2022 Mar 31;10(4):658. doi: 10.3390/healthcare10040658 (PMC9031265; doi:10.3390/healthcare10040658)
Supplement: Supplementary file 1 [file healthcare-10-00658-s001.zip › healthcare-1602326-supplementary.pdf]

Supplementary File S1: Report on accordance with the COREQ guidelines – checklist for reporting qualitative research

No Item	Description
**Domain 1: Research team and reflexivity**	
Personal Characteristics	
1. Interviewer/facilitator	Two researchers with experience in using qualitative methods conducted all focus group interviews in German and Danish (GB is a native speaker in German and speaks Danish, BRT is a native speaker in Danish).
2. Credentials	The first author and researcher GB was a Clinical Associate Professor in Palliative Care at the University of Southern Denmark, medical doctor (MD) and Master of Advanced Studies (MAS) in Palliative Care specialised in Palliative Medicine, MSA is a PhD-student, MSC is a nurse specialised in Palliative Care; HE is a medical doctor (MD) specialised in Palliative Medicine.
3. Occupation	GB was working both as a senior consultant in Palliative Medicine at the University Hospital of Southern Jutland in Sønderborg, Denmark and as a Clinical Associate Professor in Palliative Care at the University of Southern Denmark; since march 2022 he is senior consultant in Palliative Medicine and Pain therapy and the head of Palliative Medicine at the HELIOS Klinikum Schleswig, Germany. MSA is a PhD-student at the University of Southern Denmark. MSC is a nurse specialised in Palliative Care and CEO of Letzte Hilfe Deutschland gGmbH in Schleswig, Germany. HE is a medical doctor (MD) specialised in Palliative Medicine and the medical director of the Katharinenhospiz am Park gGmbH in Flensburg, Germany, an ecumenical center for hospice and palliative care, providing a palliative care unit as well as specialised palliative care for outpatients among other things. BRT was working as a researcher at the University of Southern Denmark and holds a PhD. BRT was part of the research group and conducted a number of focus group interviews but is not a co-author of the current paper.
4. Gender	GB and HE are male, MSA, MSC and BRT are female
5. Experience and training	The main investigator GB was an experienced medical doctor (MD) specialised in Anesthesiology, Palliative Medicine and had experience from research from different areas including quantitative and qualitative research and was a Clinical Associate Professor in Palliative Care at the University of Southern Denmark. He received a German doctoral degree (Dr. med.) from the University of Cologne, Germany in 2000, an Austrian Master's degree in Palliative Care from the University of Klagenfurt/IFF Vienna, and a Norwegian PhD from the University of Bergen Norway in 2017. In addition, he underwent additional formal PhD-education in Norway in both quantitative and qualitative research and medical ethics. He is one of the leaders of the Last Aid Research Group Europe (LARGE). MSA is a PhD student at the University of Southern Denmark. MSA, MSC and HE have experience with qualitative research and received supervision from GB.
Relationship with participants	
6. Relationship established	There was no relationship between the researchers/interviewers and the participants. All participants were recruited from Last Aid workshops and Last Aid courses.

7. Participant knowledge of the interviewer	The participants did get the information that the interviewers were researchers from the University of Southern Denmark and that the research aimed to explore, opinions and views of Last Aid Course participants, about the course and cultural differences in relation to care and compassion at the end of life in the border region of Germany and Denmark with minorities living on both sides of the border.
8. Interviewer characteristics	The manuscript includes information about the professional background of the interviewer. The following information is included in the methods section of the manuscript: GB was working both as senior consultant in Palliative Medicine at the University Hospital of Southern Jutland in Sønderborg, Denmark and as Clinical Associate Professor in Palliative Care at the University of Southern Denmark; since March 2022 he is senior consultant in Palliative Medicine and Pain therapy and the head of Palliative Medicine at the HELIOS Klinikum Schleswig, Germany. MSA is a PhD-student at the University of Southern Denmark. MSC is a nurse specialised in Palliative Care and CEO of Letzte Hilfe Deutschland gGmbH in Schleswig, Germany. HE is a medical doctor (MD) specialised in Palliative Medicine and the medical director of the Katharinen Hospiz am Park gGmbH, Flensburg, Germany, an ecumenical center for hospice and palliative care, providing a palliative care unit as well as specialised palliative care for outpatients among other things.

Domain 2: study design

Theoretical framework

9. Methodological orientation and Theory	The framework of the study was Palliative Care and Hospice-philosophy. The basis for the qualitative methods used was qualitative description and qualitative content analysis as described by Sandelowski, Neergaard and Malterud:<br>1. Sandelowski, M. Whatever happened to qualitative description? Res. Nurs. Health 2000, 23, 334–340.<br>2. Neergaard, M.A.; Olesen, F.; Andersen, R.S.; Sondergaard, J. Qualitative description–the poor cousin of health research? BMC Med. Res. Methodol. 2009, 9, 52.<br>3. Sandelowski, M. What's in a name? Qualitative description revisited. Res. Nurs. Health 2010, 33, 77–84.<br>4. Malterud, K. Kvalitative Metoder i Medisinsk Forskning, 3rd ed.; Qualitative Methods in Medical Research, Universitetsforlaget: Oslo, Norway, 2011.

Participant selection

10. Sampling	Purposive sampling aiming was used in the present study. All informants were participants from Last Aid workshops and courses performed within the study period from 01.01.2021 to 31.12.2021.
11. Method of approach	The participants were approached by E-mail, telephone or face-to-face by a research secretary, a researcher or facilitator of the Last Aid workshops and courses. All participants were invited to participate in the study by returning a questionnaire and/or participation in a focus group interview.

12. Sample size	People from the border regions of Denmark and Germany were invited to participate in one-day workshops and Last Aid courses with the possibility, to participate in focus group discussions after the workshop/course. Information was spread via cooperating organisations, newspapers, social media, etc, 79 people participated in Last Aid Courses and workshops. 53 of the 79 participants returned a questionnaire resulting in a return rate of 67%. 49 of the 79 participants (62%) joined one of seven focus group interviews in Danish (three focus groups) or German (four focus groups).
13. Non-participation	The number of workshops and Last Aid course participants, who chose to participate in the study is included under point 12. No informant had to be excluded from the study.
Setting	
14. Setting of data collection	The data were collected from questionnaires and focus group interviews. Informants were recruited from a group of 79 participants from eight Last Aid Courses, that were held on five different dates in the year 2021.
15. Presence of non-participants	No one else was present during the focus group interviews beside the participants and the members of the research team.
16. Description of sample	The sample is described in the methods section. The participants' characteristics are described in the result section. The informants' age ranged from 34 to 81 years with a median of 63 and a mean of 60 years of age. 41% of the informants who provided information about their age were 65 years or older whereas only 3 participants (7%) were under 40 years old. 90% of the participants were female. 30% of the informants who provided information about their profession had a medical background.
Data collection	
17. Interview guide	Information about the used introductory questions for the focus group interviews is included in the methods section.
18. Repeat interviews	No repeated interviews were carried out.
19. Audio/visual recording	All interviews were digitally recorded and stored on a computer according to the rules, regulations and recommendations of the regional Ethics Committee.
20. Field notes	All researchers could use field notes during and after the focus group interviews. The field notes included personal impressions and comments as well as other observations. Field notes were used during the analysis to question and prove the findings.
21. Duration	The duration of the focus group interviews varied from 29 to 66 minutes.
22. Data saturation	Data saturation was reached for the qualitative data from the focus group discussions, including both data saturation for the data collection and inductive thematic saturation, with a focus on analysis of the qualitative data.
23. Transcripts returned	The transcripts were not returned to the participants for comments due to practical reasons to protect the personal information and privacy. No personal information about the names or addresses of the informants was collected or stored. Therefore, it was not possible to contact the informants after the focus group interviews.

Domain 3: analysis and findings

Data analysis

24. Number of data coders	All four authors participated in data analysis and the coding process of the data.
25. Description of the coding tree	No predefined coding tree was used. The four main themes were informed by the research questions and the eight sub-themes derived from the interview data.
26. Derivation of themes	The sub-themes derived from the data and were discussed and agreed on by all authors.
27. Software	In connection to the verbatim transcription of the digital interview recordings that were done by trained secretaries, Microsoft word was used.
28. Participant checking	As explained above under point 23, there was no feedback from the participants on the findings after the analysis process in the research group. During and before the end of the focus group interviews, the interviewer gave a short summary of the interview content and asked clarifying questions. This was used as a summary of the interview themes and informant check for the findings. By using this approach the researcher could check if he/she did understand the main content and some of the important points from the interview right.

Reporting

29. Quotations presented	Themes are presented and illustrated by participant quotations that are identified by a participant number. The participant number does not correspond with the number from table 2 in order to protect the participants and to ensure confidentiality.
30. Data and findings consistent	From our point of view the presented data and findings are consistent.
31. Clarity of major themes	The major themes are presented in the results section of the paper and shown in figure 2.
32. Clarity of minor themes	Minor themes are described as part of the result chapter.
